# Macrophage phenotype and function are dependent upon the composition and biomechanics of the local cardiac tissue microenvironment

**DOI:** 10.18632/aging.203054

**Published:** 2021-05-17

**Authors:** Martin Haschak, Samuel LoPresti, Elizabeth Stahl, Siddhartha Dash, Branimir Popovich, Bryan N. Brown

**Affiliations:** 1 McGowan Institute for Regenerative Medicine, University of Pittsburgh, Pittsburgh, PA 15260, USA; 2 Department of Bioengineering, University of Pittsburgh, Pittsburgh, PA 15260, USA; 3 Department of Pathology, University of Pittsburgh, Pittsburgh, PA 15260, USA; 4 Department of Biological Sciences, University of Pittsburgh, Pittsburgh, PA 15260, USA; 5 Department of Obstetrics, Gynecology, and Reproductive Sciences, University of Pittsburgh, Pittsburgh, PA 15260, USA; 6 Clinical and Translational Science Institute, University of Pittsburgh, Pittsburgh, PA 15260, USA

**Keywords:** macrophage, cardiovascular, microenvironment

## Abstract

Macrophage accumulation and nitrosative stress are known mechanisms underlying age-related cardiovascular pathology and functional decline. The cardiac muscle microenvironment is known to change with age, yet the direct effects of these changes have yet to be studied in-depth. The present study sought to better elucidate the role that biochemical and biomechanical alterations in cardiac tissue have in the altered phenotype and functionality of cardiac resident macrophages observed with increasing age. To accomplish this, naïve bone marrow derived macrophages from young mice were seeded onto either functionalized poly-dimethyl-siloxane hydrogels ranging in stiffness from 2kPA to 64kPA or onto tissue culture plastic, both of which were coated with either young or aged solubilized mouse cardiac extracellular matrix (cECM). Both biomechanical and biochemical alterations were found to have a significant effect on macrophage polarization and function. Increased substrate stiffness was found to promote macrophage morphologies associated with pro-inflammatory macrophage activation, increased expression of pro-inflammatory inducible nitric oxide synthase protein with increased nitric oxide secretion, and attenuated arginase activity and protein expression. Additionally, exposure to aged cECM promoted attenuated responsivity to both canonical pro-inflammatory and anti-inflammatory cytokine signaling cues when compared to young cECM treated cells. These results suggest that both biomechanical and biochemical changes in the cardiovascular system play a role in promoting the age-related shift towards pro-inflammatory macrophage populations associated with cardiovascular disease development.

## INTRODUCTION

Macrophages are a heterogenous class of innate immune cells which exhibit great plasticity in polarization and function in response to various microenvironmental stimuli. Macrophage polarization is a complex process in which naïve macrophages integrate the pro- and anti-inflammatory signaling cues present within the local microenvironment to induce altered gene expression and function [1, 2]. The most well-studied of these instructive microenvironmental cues are chemical signaling cues - such as Th1 or Th2 cytokines, ECM-derived matrikines, and damage or pathogen-associated proteins (DAMPs/PAMPs) – which signal through several classes of surface receptor molecules to induce downstream transcription factor activation or deactivation [3]. Pro-inflammatory (M1) macrophage polarization states are characterized by increased phagocytosis, antigen presentation, pro-inflammatory cytokine release, and radical oxidant secretion [1, 2]. Conversely, anti-inflammatory (M2) macrophage polarization states are characterized by immunotolerant or immunosuppressive cytokine secretion, extracellular matrix remodeling, and reduced oxidant secretion [1, 2]. While chemical signaling cues play an important role in directing macrophage polarization, recent work has suggested that tissue biomechanics may also serve as an important pro- or anti-inflammatory instructive cue in naïve macrophage subsets [4–7].

Local tissue stiffness is both highly variable between organs as well as highly variable within a single organ [8]. Tissue stiffness also undergoes substantial biomechanical changes throughout an organism’s lifespan as a consequence of physiological aging, pathophysiology, and lifestyle choices such as diet or exercise [8, 9]. Modulation of tissue stiffness can have varying levels of impact on cell and organ functionality, which are often specific to the cell type and organ site in which the stiffness is changing [9]. The cardiovascular system undergoes substantial alterations in tissue biomechanics both as a function of aging as well as a function of disease-associated processes [10–12]. Functionally, cardiovascular tissue stiffening causes reduced cardiac output as cardiac stroke volume decreases due to impaired filling rates as well as attenuated cardiomyocyte contractility associated with ventricular wall stiffening [12–15]. This tissue stiffening has been shown to have significant deleterious impacts on the functionality of several cardiovascular cell populations. Increases in myocardial stiffness can reduce contractile force and action potential length in cardiomyocytes through several mechanisms [16–18]. Tissue stiffening has also been shown to induce stretch-associated Ca^2+^ channel activation with subsequent radical oxidant release, lipid peroxidation, and pro-inflammatory cytokine release in endothelial cell populations [19, 20]. In addition, tissue stiffness is known to induce the activation of resident fibroblasts, which then take on a myofibroblast phenotype and deposit excess collagen and extracellular matrix proteins in the extracellular microenvironment – further altering cardiac tissue biomechanics [21–23].

Prior studies have demonstrated a polarization response of naïve macrophages to both extracellular matrix degradation products derived from decellularized tissue as well as to culture upon differentially stiffened substrates [4–7, 24, 25]. However, the effects of aged extracellular matrix derived from decellularized cardiac tissue on naïve macrophage populations have not been investigated. Additionally, many of the previous studies examining the naïve macrophage response to differentially stiffened substrates have leveraged substrates with super-physiological elastic moduli, limiting their applicability to cardiac tissue physiology [4, 5, 7].

Thus, the present study sought to understand how age-related alterations in the composition and biomechanics of the cardiac tissue microenvironment differentially impact the phenotype and function of naïve bone marrow-derived macrophage populations. To accomplish this, a decellularization protocol optimized for cardiovascular tissue was used to remove cellular and antigenic components of young (2-4 mo) and aged (18-22 mo) cardiac tissue. Decellularized cardiac tissue was then digested and coated onto both functionalized poly-dimethyl-siloxane (PDMS) hydrogels ranging from 2 kPA to 64 kPA in stiffness, encompassing the atomic force microscopy derived stiffness values of native myocardium (~18 kPA) as well as fibrotic or pathologically remodeled myocardium (20-100 kPA), as well as onto tissue culture plastic, which exhibits an elastic modulus several orders of magnitude greater than most tissues [21, 26]. Primary bone marrow-derived macrophages were then isolated from young mice and seeded onto the coated gels. This cardiac specific *in vitro* model allows for the evaluation of the impact that biochemical and biomechanical alterations in the aging cardiovascular system have on the phenotype and function of young developmental age naïve, bone marrow-derived macrophage populations.

## RESULTS

### Cardiac extracellular matrix is effectively decellularized

**Figure 1:**
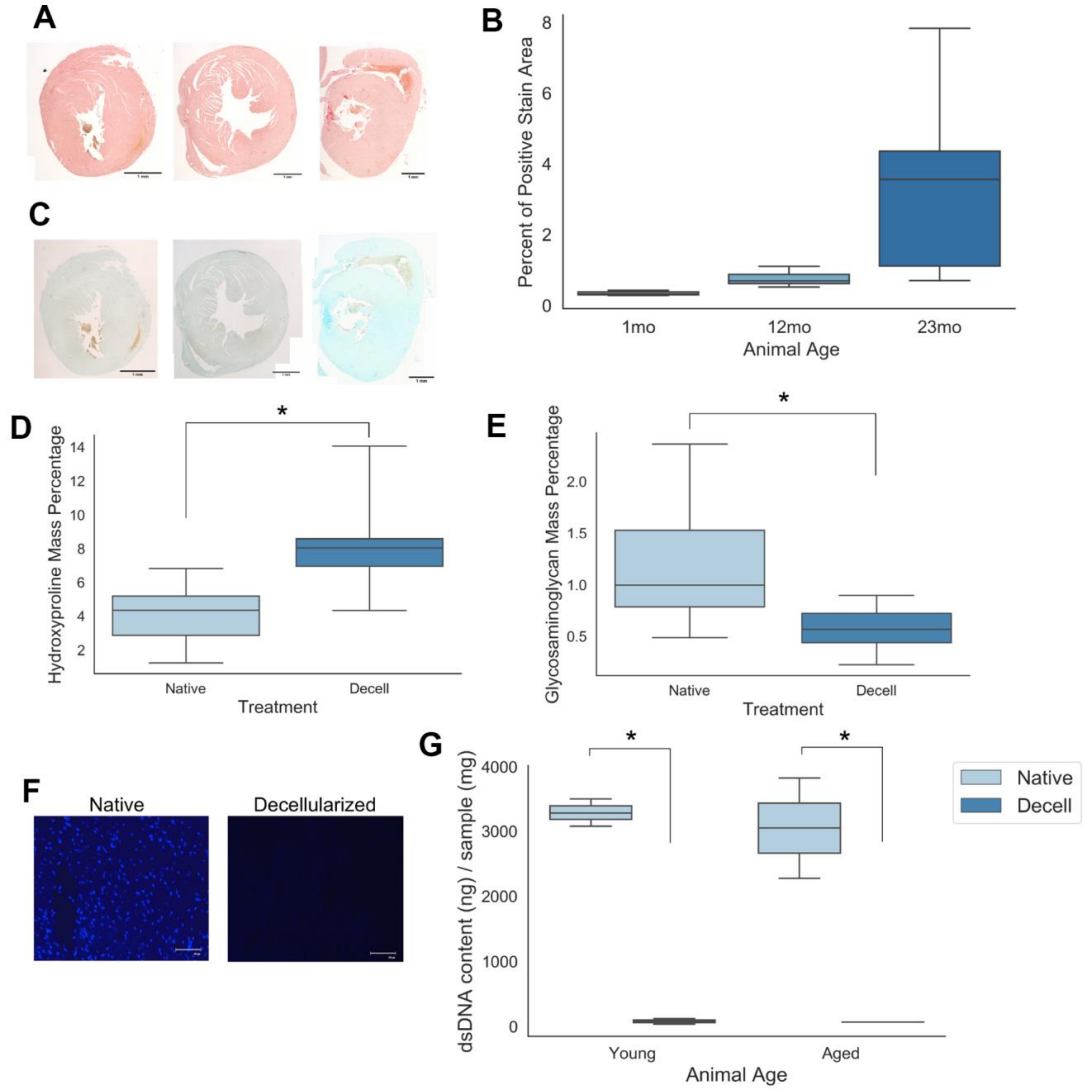
**Young and aged cardiac extracellular matrix can be decellularized while retaining matrix protein content.** (**A**) Picrosirius histological staining of collagen deposition in cardiac tissue samples isolated from mice of 1 month, 12 months, and 23 months of age. (**B**) Quantification of the percent area of positive picrosirius red expression per field of view for tissue samples from 1 month, 12 month, and 23 month old mice. (**C**) Alcian blue staining of sulfated proteoglycans in cardiac tissue samples isolated from mice of 1 month, 12 months, and 23 months of age. (**D**) Hydroxyproline mass percentage and (**E**) glycosaminoglycan mass percentage for both young and aged native as well as young and aged decellularized cardiac samples. (**F**) 4’,6-diamidino-2-phentylindole (DAPI) nuclear staining of native and decellularized tissue samples (**G**) Quantification of dsDNA content isolated from young and aged either native or decellularized cardiac tissue samples. Values shown represent mean with 25%/75%. Error bares represent standard deviation. N=4-5; * p<0.05; Scale bars (**A**, **C**) = 1mm; Scale bar (**F**) = 100um. Independent samples t-test used to assess significant trends in (**D**, **E**). One-way ANOVA with post-hoc Tukey’s test used to assess significant trends in (**G**).

To assess the impact of cardiac extracellular matrix (cECM) age on naïve macrophage responses, it was necessary to decellularize native cardiac tissue to provide a cell free scaffold which preserved matrix protein content. A skeletal muscle decellularization protocol from Wolf et al [27] with additional blood clot dissociation steps was chosen to accomplish this goal. Successful decellularization of native tissue is characterized by retention of bioactive matrix proteins while sufficiently removing DNA content and cellular antigens from the native tissue, as these factors will induce confounding immune responses in macrophages [28]. Picrosirius red staining (Figure 1A) of collagen coupled with Alcian blue staining (Figure 1C) of sulfated proteoglycans demonstrated a qualitative increase in collagen and glycosaminoglycan content with increasing age in native cardiac tissues samples. Semi-quantitative analysis of the percent positive area per field of view for picrosirius red stained cardiac sections also demonstrated a mean increase in percent positive staining area with increased sample donor age (Figure 1B). Following decellularization, samples were found to be comprised of a significantly increased mass percentage of hydroxyproline, indicative of the increased contribution of collagen content to the overall mass of the decellularized cECM scaffold (Figure 1D) following removal of the cellular content. Conversely, glycosaminoglycan mass percentage was found to significantly decrease following decellularization (Figure 1E).

The chosen decellularization protocol was found to have a significant impact on tissue nuclear content. Nuclear staining with 4′,6-diamidino-2-phenylindole (DAPI) qualitatively demonstrated a decrease in nuclear content between the native and decellularized groups (Figure 1F). Quantitatively, PicoGreen double stranded DNA quantification assay results demonstrated a significant decrease in mean double stranded DNA content between native and decellularized samples (Figure 1G). Taken together, these assay results demonstrate a qualitative and quantitative removal of nuclear content with minimal loss of matrix protein content.

### Substrate stiffness differentially impacts macrophage morphology

**Figure 2:**
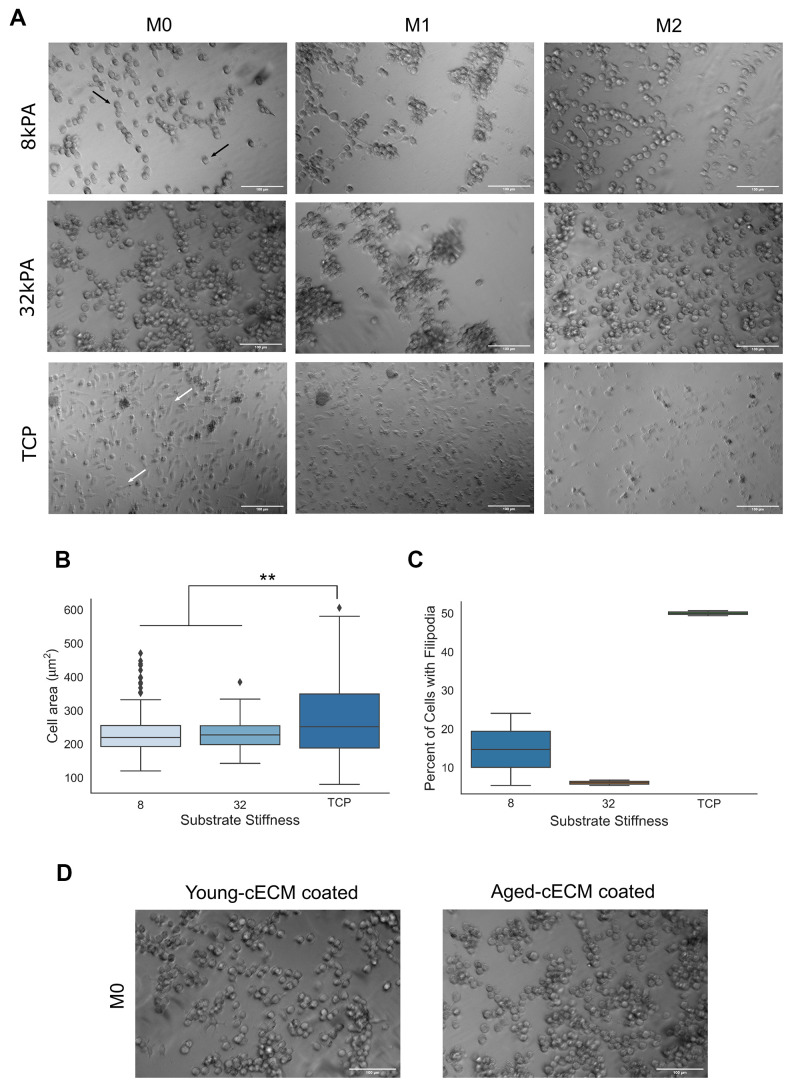
**Macrophage morphology is dependent upon the stiffness of the culture substrate.** (**A**) Naïve bone-marrow derived macrophage culture on 8kPA gel, 32kPA gel, or tissue culture plastic (TCP). Macrophages cultured on gels of lower stiffnesses tended to exhibit more round morphologies with few to no filopodial extensions (black arrows, 8kPA, 32kPA gel groups). Conversely, macrophages cultured on gels of increased stiffness, such as on tissue culture plastic exhibited more spread morphologies often with several filopodial extensions (white arrows, TCP). (**B**) Cell area was found to be significantly increased for macrophages cultured on tissue culture plastic as compared to cells cultured on gels of 8kPA or 32kPA stiffness. At least 70 cells were counted per field of view for 2-3 independent replicates. (**C**) Cells cultured on tissue culture plastic demonstrated a greater percentage of cells exhibiting filopodia per field of view. At least 70 cells were counted per field of view for 2-3 independent replicates. (**D**) Naïve bone marrow-derived macrophages cultured on 32kPA gel coated with either young (8-16wk) or aged (20-24mo), decellularized cardiac tissue displayed comparable cell morphologies. 32kPA brightfield image from 2A reproduced in 2D for qualitative comparison of the different experimental cECM coatings. TCP= tissue culture plastic. Scale bars = 100um. Data reported represents mean values with boxes representing 25%/75% of mean. Error bars represent standard deviation. Black triangles signify data point outliers. ANOVA with Tukey HSD post-hoc analysis. * p<0.05, ** p<0.001.

In order to assess the contribution of biomechanics as a predictor of macrophage morphological features, naïve bone marrow-derived macrophages isolated from young (8-16 week) mice were cultured on either 8kPA gel, 32kPA gel, or tissue culture plastic coated with decellularized cardiac extracellular matrix (cECM). Differing cell morphology was observed in response to increased culture substrate stiffness, with tissue culture plastic promoting more spread cell morphologies (Figure 2A white arrows), significantly greater cellular area (Figure 2B), and a greater percentage of cells per field of view containing filopodial extensions (Figure 2C).

In comparison, it was observed that less stiff gel culture groups tended to promote more round macrophage morphologies(Figure 2A, white arrows), significantly decreased cellular adhesion area (Figure 2B), and a decreased percentage of cells per field of view displaying filopodia as compared to macrophages cultured on tissue culture plastic (Figure 2C). In order to assess if the age of the mouse from which the cECM coating was derived affected macrophage morphology, macrophages were seeded onto 32kPA gels coated with decellularized cECM isolated from young or aged mice. It was noted that cell morphology did not appear substantially different between young or aged cECM coated gels, suggesting that substrate stiffness may be a greater predictor of macrophage morphological features than the age of the mice from which the decellularized cECM was isolated (Figure 2D).

### Stiffer substrates promote pro-inflammatory phenotype and function in naïve macrophage populations

**Figure 3:**
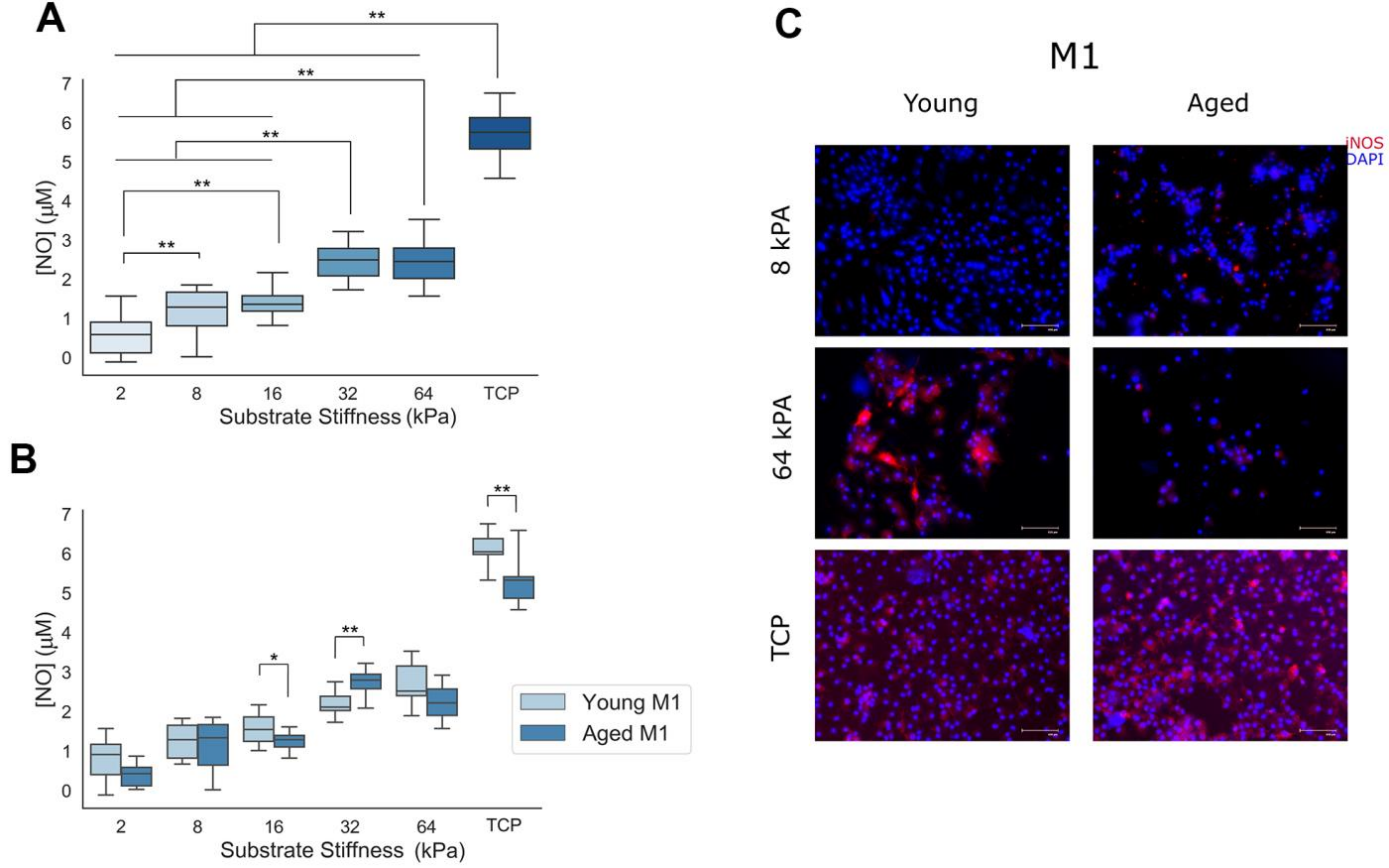
**Naïve macrophage response to pro-inflammatory M1 signaling cues is proportional to the stiffness of the culture substrate.** (**A**) Nitrite concentration in cell culture media supernatants from cells cultured on increasingly stiff substrates (n=32). (**B**) Nitrite concentration in cell culture media supernatants as a function of substrate stiffness and cECM coating age (n=16). (**C**) Representative immunolabeling of inducible nitric oxide synthase (iNOS) and cell nuclei (DAPI) in Th1 cytokine treated macrophages cultured on 8 kPA gels, 64 kPA gels, or tissue culture plastic each coated with either young or aged cECM. Representative image brightness and contrast adjusted to reduce background and highlight sample differences. TCP= tissue culture plastic. Scale bars = 100um. Data reported represents mean values with boxes representing 25%/75% of mean. Error bars represent standard deviation. ANOVA with Tukey HSD post-hoc analysis. * p<0.05, ** p<0.001.

In order to study the ability of naïve macrophages to polarize to M1 phenotypes in response to Th1 cytokine signaling cues when cultured in conditions varying in stiffness and cECM coating, macrophages isolated from young (8-16 week) mice were seeded onto cECM-coated gels ranging in stiffness from 2kPA to 64kPA or onto tissue culture plastic, and were subsequently polarized with canonical Th1 cytokine interferon gamma (IFN-γ) and lipopolysaccharide (LPS). Pro-inflammatory polarization and function was assessed through media nitrite quantification (Figure 3A, 3B) and immunolabeling for macrophage protein inducible nitric oxide synthase (iNOS) (Figure 3C).

In order to assess the significance of the observed differences, a two-way ANOVA for the independent variables gel stiffness and cECM coating age and dependent variable nitrite media supernatant concentration was performed. ANOVA results indicated a significant effect for both predictors gel stiffness (p<0.001, n=32) and cECM coating age (p<0.001, n= 92) with a significant interaction between variables noted as well (p<0.001, n=16). Tukey HSD post-hoc testing for multiple comparisons was used to assess significance between macrophage media nitrite concentrations between gels of different stiffnesses. A significant effect (p<0.001) was observed between all substrates of differing stiffnesses, save for between 8 kPA and 16 kPA (p=.348, mean diff=.2141μM) as well as between 32 kPA and 64 kPA (p=1.000, mean diff.=-.0174μM) groups. In order to assess the significance of interaction between gel stiffness and young or aged cECM gel coatings, independent samples t-tests were performed between equivalent stiffness gels coated with either young or aged decellularized cECM for each substrate stiffness tested. After correcting for multiple comparisons (Bonferroni correction – p<0.05/6=p<0.0083), a significant difference in nitrite media concentration was found between cECM coatings derived from young or aged mice for macrophages cultured on 16kPA (p=0.006, mean diff.=.3293μM), 32kPA (p<0.001, mean diff.=-.5547μM), and tissue culture plastic(TCP) (p<0.001, mean diff.=.8927μM). Qualitative immunolabeling for inducible nitric oxide synthase in fixed macrophage cultures appears to support quantified trends in macrophage media supernatant. These results indicate that expression of M1-associated protein inducible nitric oxide synthase (iNOS) and section of oxidant nitric oxide in response to Th1 stimuli is dependent upon both the stiffness of the culture substrate as well as upon the age of the host from which the decellular cECM coating was derived.

### Substrate stiffness inversely correlates with arginase activity and expression

**Figure 4:**
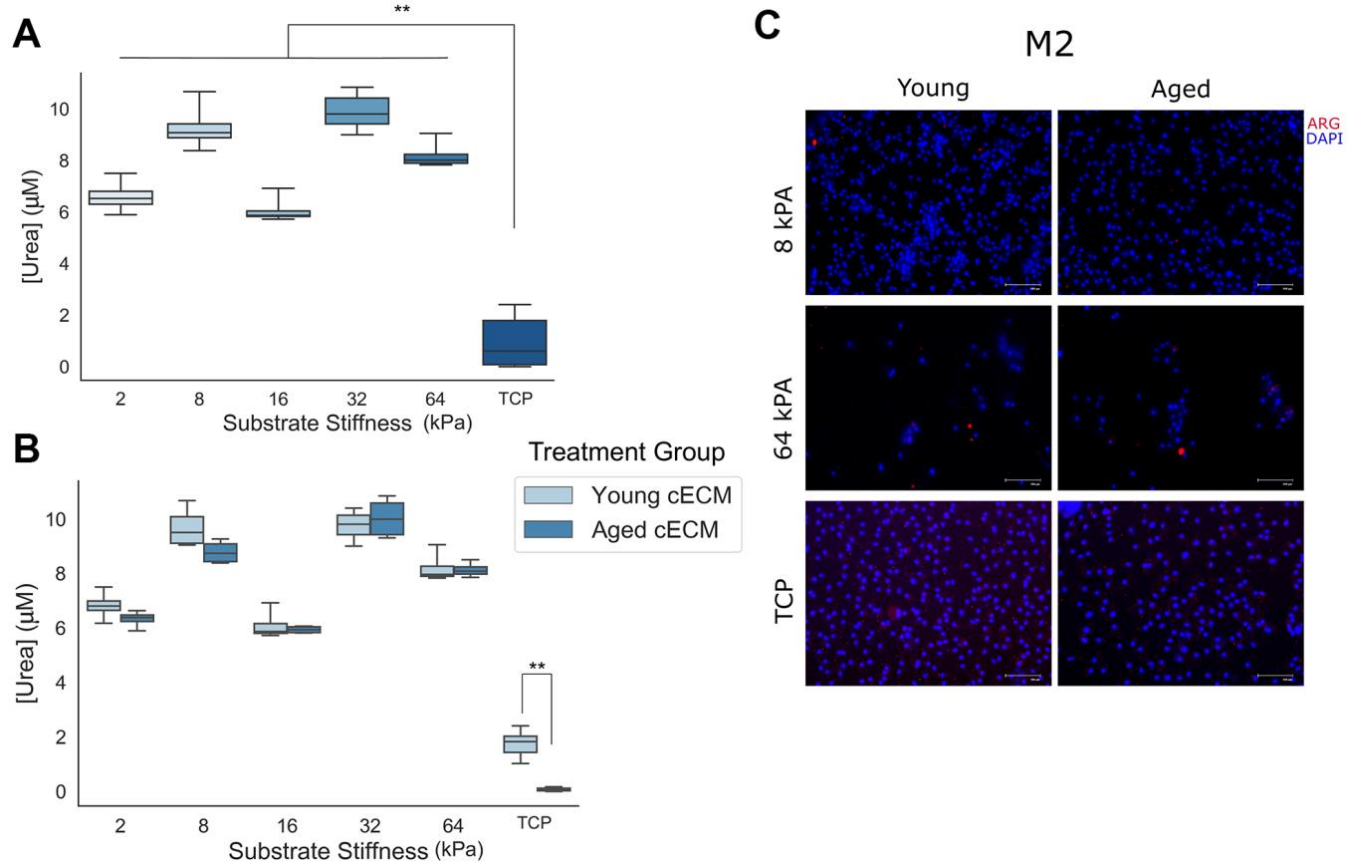
**Naïve macrophage response to anti-inflammatory M2 signaling cues is attenuated when cells are cultured on stiff culture substrates.** (**A**) Final urea concentrations in cell lysate following arginase activity assay for naïve macrophages cultured on differentially stiffened substrates (n=8-12). (**B**) Urea concentrations in cell lysates following arginase activity assay as a function of substrate stiffness and cECM coating age (n=4). (**C**) Representative immunolabeling of Arginase-1 (ARG) and cell nuclei (DAPI) in Th2 cytokine treated macrophages cultured on 8 kPA gels, 64 kPA gels, or tissue culture plastic each coated with either young or aged cECM. Image contrast adjusted to reduce background fluorescence. Scale bars = 100um. Data reported represents mean values with boxes representing 25%/75% of mean. Error bars represent standard deviation. ANOVA with Tukey HSD post-hoc analysis. * p<0.05, ** p<0.001.

In order to study the ability of naïve macrophages to polarize to M2 phenotypes in response to Th2 cytokine interleukin-4 (IL-4) when cultured in conditions varying in stiffness and cECM coating, macrophages isolated from young (8-16 week) mice were seeded onto cECM-coated gels ranging in stiffness from 2kPA to 64kPA or onto tissue culture plastic, and were subsequently polarized with canonical Th2 cytokine interleukin-4 (IL-4). Alternative macrophage polarization was then assessed through quantification of M2 macrophage function and phenotype based on expression of arginase-1, which is upregulated in murine M2 macrophages [1, 2]. Arginase function was quantified through quantification of urea production, as urea is a downstream enzymatic product of arginase (Figure 4A, 4B). Arginase-1 protein expression was assessed by immunolabeling for arginase-1 (ARG) (Figure 4C).

Urea concentration was found to exhibit variability between differentially stiffened gel macrophage cultures, which were all found to be greater than tissue culture plastic macrophage cultures. In order to assess the significance of the observed differences, a two-way ANOVA for variables gel stiffness and cECM coating age and dependent variable urea concentration was performed. ANOVA results indicated a significant effect for both predictors gel stiffness (p < 0.001, n = 8-12) and animal age from which the cECM coating was derived (p = 0.001, n = 26) with a significant interaction between variables noted as well (p = 0.001, n = 4). Tukey HSD post-hoc testing for multiple comparisons was used to assess significance between culture substrates of different stiffnesses. A significant effect (p<0.001) was observed between tissue culture plastic and all gel substrate groups. Additionally, significant differences were observed between all gel groups (p < 0.001) except for between 2 kPA and 16 kPA (p = 0.237, mean diff. = 0.5572μM) as well as between 8 kPA and 32 kPA (p=0.105, mean diff.=-0.6590μM) gels. In order to assess the significance of interaction between gel stiffness and the age of animal from which the cECM was derived, independent samples t-tests between equivalent stiffness gels coated with cECM derived from either young (8-16 week) or aged (18-21 month) mice were performed for each substrate stiffness tested. After correcting for multiple comparisons (Bonferroni correction – p < 0.05/6 = p < 0.0083), a significant effect was only observed between macrophages cultured on tissue culture plastic (TCP) coated with cECM derived from either young or aged donor cardiac tissue (p < 0.001, mean diff. = 1.670μM). Qualitative immunolabeling for macrophage Arginase-1 in fixed macrophage cultures appeared variable in expression. These results suggest that M2 polarization in response to canonical Th2 cytokine IL-4 appears to remain functional across culture groups of physiological stiffness (2kPA to 64kPA), however a significant attenuation in function was noted at supraphysiological stiffnesses, such as in the case of naïve macrophage culture on tissue culture plastic.

### cECM isolated from differentially aged animals induces altered gene expression in naïve macrophage populations

**Figure 5:**
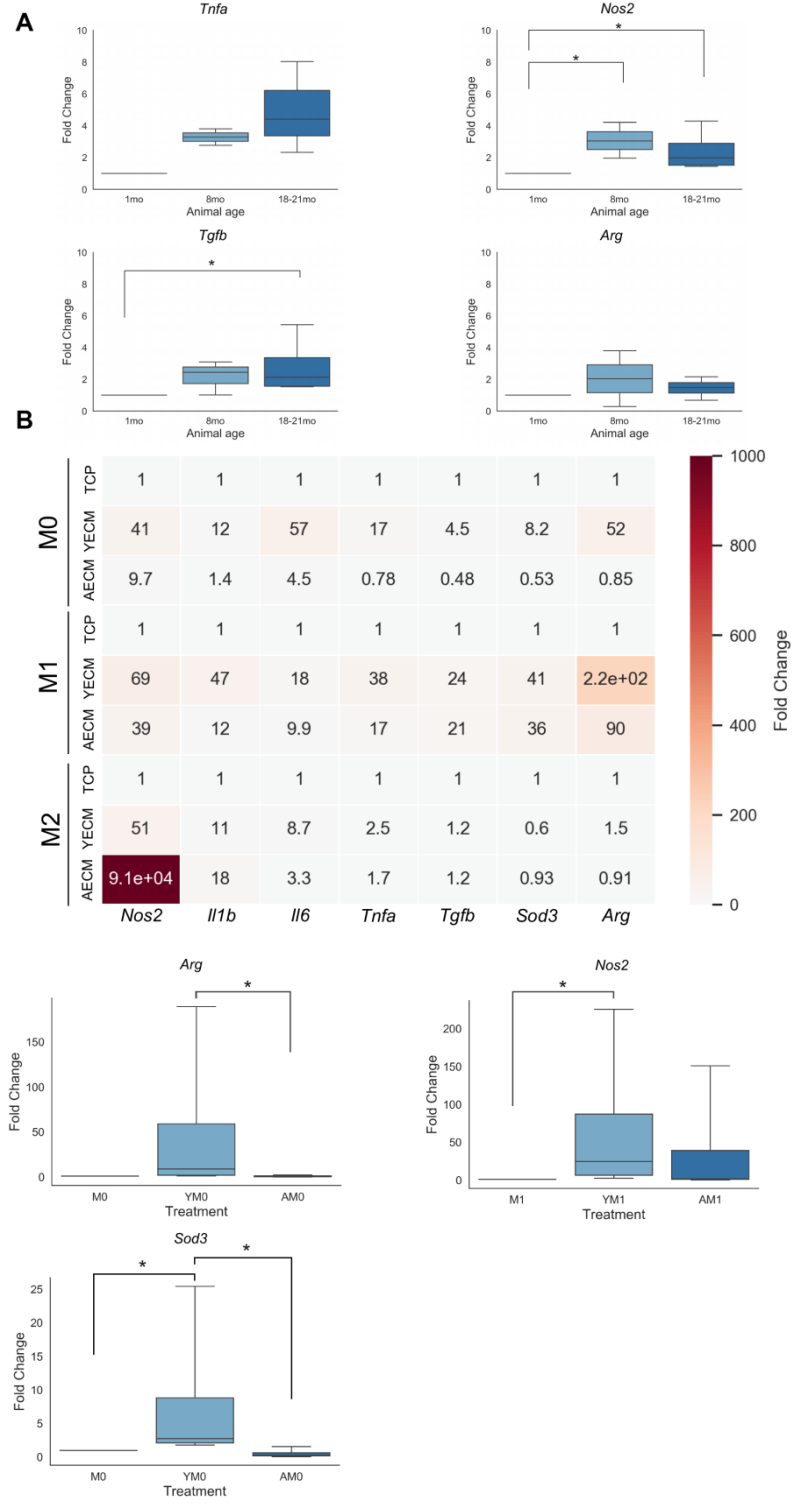
**Differentially aged decellularized cECM promotes altered gene expression patterns in macrophage populations.** (**A**) Fold change qRT-PCR results of pro- (*Nos2*, *Tnfa*) and anti-inflammatory (*Tgfb*, *Arg*) gene expression in RNA isolated from whole heart tissue lysates isolated from either young (1 month), moderately aged (8 months) or advanced age (18-21 month) C57/Bl6 mice (n=3-5). (**B**) Heatmap of qRT-PCR results for pro- and anti-inflammatory gene expression in macrophages isolated from young mice (8-16 week) cultured on tissue culture plastic coated with either young(Y, 8-16 week) or aged(A, 18-21 month) cECM with either no supplemental cytokine treatment (M0, YM0, AM0), Th1 cytokine treatment (M1, YM1, AM1), or Th2 cytokine treatment (M2, YM2, AM2) (n=4). Significant effects are noted below heatmap in boxplots with data reported as mean values with boxes representing 25%/75% of mean. Error bars represent standard deviation. Significance was assessed using a Kruskal-Wallis 1-way ANOVA with post-hoc pairwise comparisons. * p<0.05.

In order to understand how pro- and anti-inflammatory gene expression changes in aging cardiovascular tissue, RNA was isolated from whole cardiac tissue homogenates isolated from either young (1 week), moderately aged (8 months), or advanced aged (18-21 months) mice. Relative gene expression was quantified and expressed as a fold change in pro- or anti-inflammatory gene expression normalized to housekeeping control gene *Gapdh* (Figure 5A).

In native cardiac tissue, it was observed that expression of both pro- and anti-inflammatory cytokines increased expression levels with increasing age (Figure 5A). A significant difference was noted between expression levels of pro-inflammatory *Nos2* expression in RNA isolated from 8 month and 18-21 month old cardiac samples as compared to 1 month cardiac RNA isolates. A significant increase in expression of *Tgfb* was also observed in 18-21 month cardiac RNA samples relative to 1 month RNA samples.

In order to better understand if these observed differences in native tissue gene expression levels may be a consequence of age-dependent alterations in the cECM, macrophages were seeded onto tissue culture plastic with young or aged cECM, followed by either no treatment (Y→M0, A→M0), treatment with Th1 cytokine IFN-γ and LPS (Y→M1, A→M1), or treatment with Th2 cytokine IL-4 (Y→M2, A→M2). Differential gene expression determined by normalizing to control M0, M1, or M2 macrophage cell gene expression patterns was chosen to highlight alterations in macrophage polarization dynamics induced by differential cECM treatments relative to baseline expression following polarization. It was observed that young cECM treatment in general induced greater fold changes in expression levels of both pro- and anti-inflammatory factors as compared to aged cECM treatment in macrophage groups with no cytokine treatments (Figure 5B). For macrophages treated with Th1 cytokines (M1, Y→M1, A→M1), the trends in gene expression levels noted for the M0 condition were largely maintained, with young cECM tending to induce greater levels of both pro- and anti-inflammatory gene expression levels as compared to aged cECM. It was noted that young cECM induced a relatively large induction of anti-inflammatory gene *Arg* (220 vs 90). For the M2 polarization condition, treatment with aged cECM promoted a large increase in expression of pro-inflammatory *Nos2* relative to control M2 macrophage gene expression. A decrease in expression of anti-inflammatory factors arginase (*Arg*) and superoxide dismutase (*Sod3*) was observed in macrophages treated with aged cECM relative to M2 control macrophage gene expression. A significant effect was noted in the M0 treatment condition for anti-inflammatory genes *Arg* and *Sod3*. Young decellularized cECM was found to promote significantly greater mean expression of *Sod3* relative to both uncoated TCP and aged cECM coated conditions (Figure 5B). Additionally, young cECM treatment was found to induce a significant increase in macrophage *Arg* expression relative to macrophages cultured on aged cECM-coated tissue culture plastic (Figure 5B). In the M1 condition, a significant increase was also noted between macrophages cultured on young cECM-coated tissue culture plastic relative to macrophages cultured on uncoated tissue culture plastic (Figure 5B). These results suggest that macrophage culture in the presence of young cardiac extracellular matrix without polarizing cytokine cues tends to promote anti-inflammatory gene expression relative to culture on aged cECM-coated tissue culture plastic. In the presence of Th1 or Th2 cytokine cues, young cECM tends to promote enhanced expression of appropriate canonical M1 or M2 macrophage genes while aged cECM culture tended to attenuate macrophage gene expression levels in response to cytokine cues.

## DISCUSSION

The results of this study demonstrated how macrophage culture upon substrates of varying stiffness coated with cECM derived from either young or aged murine donors is able to influence both pro- and anti-inflammatory macrophage polarization. Macrophages cultured on gels of lower elastic modulus, such as 2 kPA or 8 kPA, tended to exhibit round morphologies with fewer filopodial extensions, a morphology which has been shown to be associated with alternatively activated macrophage populations [6]. Additionally, macrophage culture on lower elastic modulus substrates attenuated expression of pro-inflammatory inducible nitric oxide synthase, reduced concentrations of nitrite in macrophage culture supernatant, and enhanced arginase activity relative to highly stiffened groups. Conversely, naïve macrophage culture on substrates of greater elastic modulus progressively promoted morphological changes characterized by more spread morphologies often with several filopodial extensions, a morphology reported to be associated with macrophages treated with LPS and IFN-γ [6]. Additionally, quantification of nitrite in macrophage culture media supernatant from higher elastic modulus culture groups demonstrated an enhanced functional pro-inflammatory response which was supported qualitatively by iNOS immunolabeling. While arginase activity was variable between differential stiffness gel culture groups, a significant attenuation in arginase activity was observed between all gel groups and the tissue culture plastic culture group. These results confirm the previously reported mechanoresponsivity of naïve macrophage populations characterized by increasingly pro-inflammatory phenotypes when cultured on substrates of increasing elastic modulus. Our study also builds upon these results to demonstrate an effect at physiologically relevant ranges of tissue stiffness, including at the range of observed cardiac tissue stiffening between native and pathological cardiac tissue [6, 7, 26]. It additionally confirms the previously reported pro-inflammatory phenotypic biasing of macrophage populations when cultured upon highly stiff substrates such as plastic or glass, particularly when cultured in the presence of exogenous Th1 cytokine polarization cues [4, 5]. It is important to note that some of the greatest changes in macrophage phenotypic and functional responses were altered following naïve bone marrow-derived macrophage culture on tissue culture plastic substrates, an effect which can most likely be attributed to the large order of magnitude difference in stiffness between the experimental gel groups and the tissue culture plastic, which can exhibit elastic moduli greater than 10MPa.

The present study also identified cardiac extracellular matrix age as a predictor of naïve macrophage phenotype and function. Aged cardiac extracellular matrix was observed to decrease both pro- and anti-inflammatory function, albeit to differing extents, with a mean reduction of 12% and 9.7% in the concentrations of nitric oxide and urea, respectively. The reduced M1/M2 functionality following aged cECM treatment was also supported by the gene expression trends quantified by qRT-PCR. Significant increases in relative expression of anti-inflammatory genes *Arg* and *Sod3* were observed following culture with cECM derived from young (2-4 month) mice in the M0 condition, suggesting that without exogenous polarization cues cECM derived from young animals promoted alternatively activated macrophage gene expression. Additionally, a significant increase in *Nos2* expression was observed in macrophages when cultured with cECM derived from young mice in the presence of Th1 cytokines. Taken together these results suggest that young cardiac extracellular matrix promotes alternative activation in the absence of cytokines but also supports macrophage polarization and activation when cultured in the presence of Th1 or Th2 cytokines. Conversely, macrophage culture with cECM derived from aged mice attenuated the increased expression of anti-inflammatory gene expression in the M0 control culture, attenuated pro-inflammatory gene upregulation in response to Th1 cytokines, and induced non-canonical upregulation of pro-inflammatory genes such as *Nos2* or *Il1b* in the presence of alternative activation promoting cytokine IL-4. However, it is important to note that this trend was not observed for all pro-inflammatory gene targets assayed, such as in the case of *Il6 or Tnfa.* It is also important to note that the observed transcriptional alterations induced by decellularized cardiac extracellular matrix were only characterized at a high level using canonical M1 or M2-associated genes and future studies should seek to further expand upon the insight derived from this study by using a more comprehensive transcriptional landscape characterization tool such as RNA sequencing. Additional insight into the mechanism underlying these observed results may also be elucidated by epigenetic or other chromatin accessibility assays.

When examining the relative contribution of biomechanics and biochemical changes as a determinant of macrophage function, the data derived from the present study implicate biomechanical alterations within the cardiovascular system as the principle mechanism which promotes pro-inflammatory phenotypes and attenuation of anti-inflammatory function in naïve macrophage populations. For example, macrophages cultured on gels of lower stiffness consistently exhibited lower nitric oxide production as compared to gels of increased stiffness or on tissue culture plastic, regardless of cardiac ECM coating age. While biomechanics were implicated as the primary determinant of macrophage phenotype and functionality in the present study, it is important to note that the *in vitro* model utilized may not fully reproduce the biochemical alterations which occur *in vivo,* nor does it reproduce the mechanisms by which these changes may occur*.* Additionally, while this study was able to decouple the variables of biomechanics and biochemistry, these factors are inherently linked *in vivo* with organ composition and structure being the mechanism through which material stiffness is conferred to the cardiovascular system.

These findings have several important implications for both biomaterial design as well as for the application of cardiac extracellular matrix-derived therapeutics. First, the results of the present study highlight the importance of matching tissue elastic modulus in therapeutics which do not seek to induce excessive macrophage activation and potential chronic inflammatory signaling in the case of non-degradable material implants, as a timely re-polarization of macrophages from a predominantly M1 response to a more alternatively activated M2 population is a hallmark of functional biomaterial integration [2]. Additionally, the significant reduction of naïve macrophage priming observed in aged cECM treatment coupled with the tendency of aged cECM to upregulate pro-inflammatory gene transcripts following IL-4 treatment suggests that aged cECM may be a less instructive material relative to younger source age cECM therapeutic products and may promote chronic inflammatory processes even in the presence of anti-inflammatory signaling cues. Interestingly, excessive radical oxidant secretion through the inducible nitric oxide synthase pathway has also recently been implicated as a mechanism for the acquisition of the heart failure with preserved ejection phenotype, with macrophages playing an important role in promoting cardiac diastolic dysfunction [29, 30]. The results of this study implicate matrix stiffening as a potential mechanism for the macrophage mediated acquisition of diastolic dysfunction through the increasing promotion of radical oxidant secretion through the inducible nitric oxide synthase pathway in aging populations. Additionally, the reduction in the ability of aged cardiac extracellular matrix to polarize macrophages both at baseline and in the presence of canonical Th1 or Th2 cytokine cues suggests that the aging cardiac microenvironment may promote chronic cardiac inflammatory processes through both an increase in pro-inflammatory macrophage phenotype and function induced through exposure to stiffened cardiac tissue as well as through a reduced ability to shift macrophage polarization towards constructive phenotypes following inflammatory phenotypic polarization.

However, it is also essential to note that the present model includes only a fraction of intercellular and matrix-cell interactions present within the cardiovascular system. While cardiac macrophages play an important role in maintaining functional homeostasis and the tissue reparative response following cardiac tissue damage, it is also essential to consider the impacts that biochemical and biomechanical alterations have on the other constitutive cells of the cardiovascular system, including cardiomyocytes [16, 31], endothelial cells [32], fibroblasts [21, 23], and circulating progenitor cells [33]. *In vivo*, the changing cardiovascular microenvironment induces altered cell functionality, paracrine, and endocrine signaling, which then not only alters the local tissue composition and biomechanics but also induces systemic changes within the organism, such as increased blood pressure, decreased tissue oxygen saturation, reduced distal tissue perfusion, and kidney dysfunction [34]. These systemic changes can also cause alterations in distal tissue microenvironments, altering the function of the constitutive cells of that organ, and biasing the secretome of those cells towards the secretion of increasingly pro-inflammatory factors and chemokines; which can in turn contribute to the progression towards cardiac tissue dysfunction and the “inflammaging” phenotype commonly observed in aged individuals [1, 34]. Thus, while the results described in this manuscript provide important insights into how the biomechanical and biochemical changes which occur in the aging cardiac microenvironment promote pro-inflammatory macrophage phenotypic and functional responses, this model does not approach fully recapitulating the complexity of the microenvironment or the numerous intercellular paracrine and endocrine signaling pathways which exist in these microenvironments *in vivo*. Additionally, without inclusion of additional cardiac cell populations, it can be difficult to assess if the macrophages directly contribute to cardiac dysfunction or whether the effect is primarily mediated through paracrine and endocrine signaling factors released into circulation by macrophages which act upon other cell populations which have been shown to be important predictors of cardiovascular health in the aged, such as CD34+ circulating endothelial progenitor cell populations [32, 33, 35]. Future studies can expand upon the applicability of this model for understanding the mechanisms underlying cardiovascular disease by introducing an *in vitro* co-culture model using additional resident cardiac cell populations coupled with a relevant *in vivo* model of cardiac microenvironmental alteration to better recapitulate the complexity of cardiac intercellular and cell-matrix interactions.

There are several additional limitations of the present study that merit further investigation to elucidate the roles that biomechanics and biochemistry play in dictating macrophage phenotype and functionality. First, the present study was only able to provide a limited characterization of the differences between the young and aged cardiac extracellular matrix both prior to and following decellularization. Previous work by Williams et al. has characterized significant changes in matrix protein content by mass spectrometry between neonatal, perinatal, and adult cardiac tissue which has a significant differential impact on cardiomyocyte proliferative potential, with a general trend towards an accumulation of fibrillar collagen subtypes with increasing age [36, 37]. A similar characterization should be a future direction of study to better understand how matrix protein content is altered between young and aged populations, particularly with a focus on quantifying these differences in matrix protein content in young and aged samples with mass spectrometry or other analytic chemistry assays. Additionally, future studies should seek to better understand how these matrix protein content alterations which occur with increasing age in turn alter the biomechanical properties of the aging cardiac microenvironment. The experimental model can also be expanded in future studies to include decellularized cardiac extracellular matrix coating derived from pathological cardiac tissue to further extend the applicability of the described *in vitro* model and help further develop mechanistic insight. Finally, the present study focused on the impact of biochemical and biomechanical changes on naïve bone marrow-derived macrophages; however, this only represents one population of macrophages present in the adult and aging myocardium. Yolk sac derived cardiac macrophages also represent a homeostatically important macrophage population within the adult myocardium and undergo significant change with increasing age [38, 39]. Elucidation of the alterations in phenotype and function which occur in this macrophage population in response to changes in matrix biochemistry and biomechanics will be important to fully understand how changes in these variables propagate throughout the cardiac macrophage compartment and contribute to the age-related risk of cardiovascular disease pathogenesis. Additional studies could also work towards developing models which use resident macrophages isolated from cardiac tissue with flow cytometry and reintroduced into the various culture conditions to better understand the impacts of biochemical and biomechanical alterations on the resident macrophage populations.

In summary, the present study sought to elucidate how two principle variables of change within the aging cardiovascular system – biomechanical and biochemical changes – impact naïve, young bone marrow-derived macrophage populations using a physiologically relevant *in vitro* model of cardiovascular aging. Results of the study identified a significant effect for both predictors as well as the interaction of these variables in some conditions, with increased gel stiffness promoting cell morphologies associated with pro-inflammatory macrophage populations, increased inducible nitric oxide synthase protein expression coupled with enhanced nitric oxide secretion, and attenuated anti-inflammatory arginase activity. Additionally, it was observed that differentially aged, decellularized cardiac ECM induced less of a priming response relative to young cardiac ECM in the M0 and M1 conditions, and promoted increased expression of pro-inflammatory gene transcripts with a minor reduction in anti-inflammatory gene expression relative to young cardiac extracellular matrix following M2 stimulus. These results elucidate the role that two principle variables of change in the aging cardiovascular system play in promoting the shift towards a more pro-inflammatory macrophage phenotype and functionality with an attenuation of responsivity to Th2 cytokine signaling cues with increasing age in naïve bone marrow-derived macrophage populations. Future studies will focus on further developing the mechanistic insights derived from the results of this study by developing relevant *in vivo* models of cardiac microenvironmental change as well as expanding the scope of the described *in vitro* model to include co-culture of macrophages with other resident cardiac cell populations as well as culture of cardiac resident macrophage populations.

## MATERIALS AND METHODS

### Animal care and use

All animal experiments were approved by the Institutional Animal Care and Use Committee at the University of Pittsburgh. Young wild type C57BL/6 mice were obtained from Jackson Labs (Bar Harbor, ME) and bred in a pathogen-free rodent facility for use in the described experiments. Eighteen to twenty-two month old C57BL/6 mice were obtained from the National Institute of Aging (NIA) aged rodent colony (Charles River, Wilmington, MA). Animals were housed according to the University of Pittsburgh guidelines in a pathogen free facility with 12 hr / 12 hr light to dark cycle with food and water *ad libitum*. Mice utilized for experiments were sacrificed by carbon dioxide inhalation followed by cervical dislocation, in accordance with Institutional Animal Care and Use Committee guidelines.

### Decellularization of cardiac tissue

The decellularization protocol used in the present study is based on a skeletal muscle decellularization protocol first described by Wolf et al. with several modifications [27]. Briefly, young (8-16 week old) and aged (20-24mo) cardiac tissue was isolated from C57BL/6 mice (Jackson laboratories, NIA), cleaned to remove any connective tissue or vasculature removed during isolation, and washed with 1x PBS three times to wash blood content out of cardiac tissue. Samples were then minced and incubated in streptokinase (Sigma, St. Louis, MO) at 1000 IU/mL overnight at room temperature to remove any additional clotted blood from cardiac tissue. Samples were washed twice in 1x PBS. Cardiac tissue samples were then placed in a 2:1 v/v chloroform:methanol solution and were shaken on an orbital shaker at 300 rpm for 2 hours. Cardiac tissue then went through a graded series of ethanol (100, 90, 70, 50, 0) for 30 minutes each under constant shaking at 300 rpm. Samples were then incubated at 37° C in 0.2% Trypsin / 0.2% EDTA solution for 2 hours. Samples were washed with deionized water followed by 2x PBS for 30 minutes each at room temperature under constant agitation at 300rpm. Samples were then shaken at 300rpm with 2% (w/v) sodium deoxycholate (Sigma, St. Louis, MO) for 5 hours at room temperature, washed with deionized water for 30 minutes, washed with 2x PBS for 30 minutes, and shaken in 2% sodium deoxycholate overnight for 14-16 hours. Samples were subsequently shaken at 300rpm in 1% Triton X-100 (Fisher, Hampton, NH) for 1 hour followed by a 30 minute wash in deionized water. Samples were then shaken at 300 rpm in a 0.1% (w/v) peracetic acid (Sigma, St. Louis, MO) / 4% (v/v) ethanol solution for 2 hours to sterilize the decellularized cardiac tissue. Decellularized tissue samples were then washed twice in 1x PBS and deionized water for 30 minutes per wash. Decellularized cardiac tissue was then frozen in Type I water and lyophilized. Lyophilized cECM was then split for either DNA quantification and biochemistry quality control assays or hydrogel coating and cell treatment.

### cECM DNA isolation

Lyophilized decellularized cardiac tissue samples were digested in proteinase K digest buffer (0.5% proteinase K (Thermo, Waltham, MA) in 10mM Tris-HCl, 100mM NaCl, 25mM EDTA, and 0.5% sodium dodecyl sulfate made up in Type I water) at a concentration of 50 mg cECM/mL proteinase K digest buffer at 50° C under agitation for 24 hours or until no visible tissue fragments remain. After full sample digestion, an equal volume of a 25:24:1 (v/v/v) phenol/chloroform/isoamyl alcohol was added to each sample. Samples were mixed by inversion and were then centrifuged at 10,000g for 10 minutes at 4° C. Following centrifugation, the aqueous fraction of the solution containing any DNA present in the sample was carefully removed from the top of the solution. Care was taken to avoid the uptake of any precipitate formed at the aqueous/organic phase interface. Fractionation and aqueous phase collection was repeated until no visible precipitate formed at the aqueous/organic phase interface of the solution. A 1/10^th^ sample volume of 3M sodium acetate and 2x sample volume of 100% ethanol was then added to the aqueous phase and sample tube was mixed by inversion several times. Samples were then placed in dry ice until fully frozen, after which samples were centrifuged at 10,000g at 4° C for 10 minutes. Any DNA isolated from decellularized cECM samples will pellet out during centrifugation. Supernatant was carefully decanted off sample tubes and DNA isolates were washed with 70% ethanol and centrifuged at 10,000g for 10 minutes at 4° C. Ethanol wash supernatant was decanted and samples were spun again at 10,000g for 1 minute at 4° C. Any residual ethanol was pipetted out with care not to disturb the DNA pellet.

### Doubled stranded DNA quantification

PicoGreen double stranded DNA (dsDNA) quantification kit (Thermo, Waltham, MA) was used for quantification of dsDNA isolated from native and decellularized cardiac samples. dsDNA standard solution was prepared from a 2,000 ng/mL stock and serially diluted in TE buffer. Isolated DNA samples were resuspended in 1mL of 1X TE buffer for 5 minutes at room temperature under gentle agitation. Samples were then serially diluted in 1X TE buffer. One hundred microliters of each sample and dsDNA standard was plated on a 96 well plate. One hundred microliters of Quant-iT PicoGreen reagent was added to each standard or sample and was incubated in the dark at room temperature for 5 minutes, after which time fluorescence emission was measured using a Synergy HTX plate reader (BioTek, Winooski, VT) using an excitation wavelength of 480 nm, an emission wavelength of 520 nm, and a cutoff wavelength of 515 nm. dsDNA concentration for each sample was calculated from the equation derived from the linear regression of dsDNA standard fluorescence emission vs. standard dsDNA concentration. The sample dilution whose reading was closest to the center of the standard curve was used for calculating sample dsDNA content, and calculated dsDNA content was multiplied by the dilution factor to determine accurate concentrations.

### Tissue histology

Native cardiac tissue samples were fixed in 10% neutral buffered formalin, embedded in paraffin, and sectioned by the McGowan Institute for Regenerative Medicine Histology Core. Tissue sections were deparaffinized by xylene washes, followed by a graded series of ethanol washes, followed finally by tap water wash. Tissue sections were then stained with either picrosirius red to visualize collagen or alcian blue for sulfated proteoglycan visualization. Brightfield imaging of stained sections was performed with Nikon Eclipse50i (Nikon, Melville, NY). Tissue section images were stitched together to form composite images using Photoshop (Adobe, San Jose, CA). Semi-quantitative analysis of the percent area of positive staining in picrosirius red images was performed using NIH FIJI biological imagining analysis software (National Institutes of Health, Bethesda, MD).

### Papain digest

Lyophilized decellularized cardiac extracellular matrix samples were massed to obtain dry weight prior to digestion. The papain digest solution was prepared by adding 0.125 mg papain / mL (Sigma, St. Louis, MO) and 0.10M L-cysteine (Sigma, St. Louis, MO) to phosphate-buffered EDTA (PBE) buffer (0.1 M Na_2_HPO_4_, 0.01 M Na_2_EDTA, pH 6.5). Samples were digested in 1 mL of papain digestion solution and incubated at 60C for 12-16 hours or until samples were fully digested. Following complete digestion, samples would be split for either hydroxyproline quantification or glycosaminoglycan quantification.

### Hydroxyproline assay

A 500 μg/mL stock solution of hydroxyproline (Sigma, St. Louis, MO) was prepared in PBE buffer and serially diluted to create assay standards. Fifty microliters of each sample and standard was added to a screw-top microcentrifuge tube. Fifty microliters of 2M NaOH was added to each tube and tops were secured. Samples and standards were incubated at 110° C for at least 18 hours. Tubes were then centrifuged and 10.5 μL of Type I water was added to each sample. One hundred microliters of 0.01M CuSO_4_, 100 μL of 2.5M NaOH, and 100 μL of 6% H_2_O_2_ were added sequentially to each tube and tubes were vortexed well after each addition. After 6% H_2_O_2_ addition, tubes were incubated at room temperature until samples and standards turned clear or blue in color. Samples and standards were vortexed well and incubated at 80° C for 5 minutes. Tubes were briefly centrifuged after which tubes were placed in the -80° C freezer for 5 minutes. Four hundred microliters of 3N H_2_SO_4_ was then added to each tube. Samples and standards were allowed to cool to room temperature. Two hundred microliters of a 5% dimethylaminobenzaldehyde (Sigma, St. Louis, MO) in propanol solution was added to each tube and tubes were incubated in heat block at 70° C for 15 minutes. Standards and samples were vortexed well and 200 μL of each was plated in triplicate in a 96 well plate. The absorbance at 540nm for each standard or sample well was measured using a Synergy HTX plate reader (BioTek, Winooski, VT). Experimental sample hydroxyproline content was calculated using the equation derived from the linear regression of standards’ absorbances values.

### Glycosaminoglycan assay

A 500 μg/mL standard of chondroitin-6-sulfate (Sigma, St. Louis, MO) was prepared in PBE buffer (0.1 M Na_2_HPO_4_, 0.01 M Na_2_EDTA, pH 6.5). The 500 μg/mL standard was then serially diluted in PBE buffer to generate subsequent standards for curve fitting. Fifty microliters of each standard and papain-digested sample was pipetted into a 96 well plate in triplicate. Dimethylmethylene blue (DMMB) solution was prepared by dissolving 1 mg of 1,9-dimethylmethylene blue zinc chloride double salt (Sigma, St. Louis, MO) in 0.313 mL of 100% ethanol with 0.148 g NaCl and 0.19 g glycine added and then diluted up to 50 mL in Type I water. Two hundred fifty microliters of DMMB solution was added to each standard or sample well. Standard and sample absorbance at 525 nm was measured using a Synergy HTX plate reader (BioTek, Winooski, VT). Sample glycosaminoglycan content was calculated using the equation derived from the quadratic regression of standards’ absorbance values.

### PDMS gel coating

Decellularized young and aged cardiac extracellular matrix was pepsin-digested at a concentration of 10 mg/mL for 48 hours. Young and aged cECM digests were then diluted down to a concentration of 1 mg/mL with 1X PBS, neutralized to 6 pH, and incubated with sterilized, functionalized polydimethylsiloxane gels of either 2 kPA (Advanced Biomatrix, San Diego, CA), 8 kPA (Advanced Biomatrix, San Diego, CA), 16 kPA (Advanced Biomatrix, San Diego, CA), 32 kPA (Advanced Biomatrix, San Diego, CA), or 64 kPA (Advanced Biomatrix, San Diego, CA) for 1 hour at room temperature. Following incubation, cECM digests were aspirated off of gels and gels were washed three times with 1X PBS. The final wash in 1X PBS was left on the gel to prevent any gel drying per manufacturer’s instructions prior to cell seeding.

### Macrophage isolation, culture, and seeding

The tibia and femur were isolated from young (8-16 week) C57BL/6 mice (Jackson, Bar Harbor, ME) and all muscle and connective tissue was separated from the bone. Clean bones were then washed in macrophage culture media (Dubelco’s modified eagle medium, 10% fetal bovine serum, 1% HEPES, 1% Pen/Strep, 2% non-essential amino acids, and 10% L929 fibroblast culture supernatant) to remove any adherent tissue or debris. Tibias and femurs were then transferred to a sterile hood where the epiphysis of each bone was removed. 10mL of sterile macrophage culture media was then flushed through the diaphysis of each bone to collect murine bone marrow. Care was taken to ensure that all marrow was flushed from femur and tibia prior to discarding the bones. Bone marrow-media suspensions were pipetted up and down 5x to break up any cell clumps within the marrow isolated and then passed through a 100um cell strainer to remove any residual debris and further break up any cell clumps. Marrow suspensions were then centrifuged at 1500rpm for 5min, after which the culture media was decanted off and cells were resuspended in fresh macrophage culture media. Cells were counted and plated at a density of 1x10^6^ cells/mL in petri dishes with approximately 1.5x10^7^ cells per petri dish. After 7 days of culture, adherent cells were lifted from the plates by incubating in accutase (Fisher, Waltham, MA) for 5 min, after which cells were washed off the petri dishes, counted, and re-plated onto either the cECM coated hydrogels or onto cECM coated tissue culture plastic at a density of 1x10^6^ cells/mL in macrophage culture medium. After 24 hours, cells received either no treatment, treatment with Th1 cytokines IFN-g (20 ng/mL, Peprotech) and LPS (100 ng/mL, Sigma), or treatment with Th2 cytokine IL-4 (20 ng/mL, Peprotech) for 24 hours. Following cytokine treatment for 24 hours, media was collected from experimental wells to measure nitrite production. Cells in remaining wells were split and either fixed in 2% paraformaldehyde (Fisher Scientific) for 30 minutes for immunofluorescent staining or lysed for arginase activity assays.

### Macrophage morphological imaging and quantification

To identify differences in macrophage morphological features induced by culture on substrates of differing stiffness or by substrate coating with cECM isolated from young or aged individuals, brightfield images of fixed cells were acquired with a 20x objective on a ZEISS Axio Observer microscope (ZEISS, Oberkochen, Germany). Cell area measurements were quantified with NIH FIJI biological imagining analysis software (National Institutes of Health, Bethesda, MD). Additionally, the percentage of cells exhibiting filopodial extensions were counted per image, with at least 75 cells being analyzed per field of view.

### Macrophage immunofluorescence

After fixation, cells were washed three times for 5 minutes each with 1X PBS and stored in 1X PBS at 4C until ready for immunofluorescence staining. Cells were blocked in donkey blocking buffer (5% donkey serum (Fisher Scientific), 1% bovine serum albumin (Sigma), 0.1% Tween-20-Triton X-100 (Fisher Scientific)) for 1 hour at room temperature. Following block, cells were stained with primary antibody, either rabbit anti-iNOS (ab3523, Abcam, Cambridge, UK) diluted 1:100 in donkey blocking buffer or goat anti-liver arginase (ab91279, Abcam) diluted 1:50 in donkey blocking buffer, for 14-16 hours in the dark at 4° C. Cells were then washed three times with 1X PBS for five minutes per wash. Cells incubated with rabbit anti-iNOS primary were incubated with donkey anti-rabbit Alexa Fluor 594 secondary antibody (ab150064, Abcam) diluted 1:200 in donkey blocking buffer. Cell incubated with goat anti-liver arginase were incubated with donkey anti-goat Alexa Fluor 594 (ab150136, abcam) diluted 1:200 in donkey blocking buffer. Cells were incubated with secondary antibody for 1 hour at room temperature in the dark. Cells were washed three times with 1X PBS for five minutes per wash. Cells were then incubated with 4′,6-diamidino-2-phenylindole (DAPI) nuclear stain (Biolegend 422801, San Diego, CA) for 1 minute in the dark at room temperature. Cells were washed five times for 5 minutes per wash in 1X PBS. Cells were stored in PBS in the dark at 4° C until ready for imaging. Immunofluorescence images were captured using a FLoid cell imaging station (Thermo Scientific, Waltham, MA). Acquisition settings were set at: blue laser intensity=30%, red laser intensity=50%, optical zoom=0%.

### Nitric oxide quantification

Standards were made from a 0.1M NaNO_2_ stock solution mixed with 1 mL of macrophage media which was subsequently serially diluted. 50uL of both standards as well as media collected from experimental groups wells was plated in triplicate in a 96 well plate. Fifty microliters of sulfanilamide (Sigma, St. Louis, MO) was added to each well and plates were incubated in the dark for 10 min at room temperature. Fifty microliters of N-(1-Naphthyl)ethylenediamine (Sigma, St. Louis, MO) was pipetted onto each well and plates were incubated in the dark for 10 min at room temperature. Following N-(1-Naphthyl)ethylenediamine treatment, cells were transferred to Synergy HTX plate reader (BioTek, Winooski, VT) and sample absorbance at 540 nm was measured for each sample and standard. Sample nitrite concentration were calculated from the linear equation derived from the linear regression of serially diluted nitrite standards.

### Arginase activity quantification

Cells were lysed in lysis buffer composed of 0.1% Triton X-100 and 1% 100X Halt Protease Inhibitor (Themo Scientific, Waltham, MA) in Type I water. 50uL of lysis buffer was added per well to ensure sufficient protein concentrations. Cells were incubated with lysis buffer for 15min at room temperature. Following lysis, 25 μL of cell lysate was pipetted into a new 96 well plate and 25 μL of arginase activation solution (10 mM MnCl_2_ in 50 mM Tris buffer, pH 7.5) was added to each sample. Samples were then covered and incubated at 55° C for 10 minutes. Samples were removed from the incubator and 50 μL of L-arginine (Sigma, St. Louis, MO) substrate solution was added to each well, after which samples were incubated for 2 hours at 37° C. To determine relative arginase activity, it is necessary to measure the concentration of urea in each well following arginase activation and incubation with substrate solution. To accomplish this, urea standards were made up at 100 mg/mL in lysis buffer and serially diluted to create standard curve. To account for dilutions of experimental samples during sample preparation, 75 μL of Type I water was added to each standard. Following sample and standard preparation, 20 μL of each was pipetted into a new 96 well plate and 200 μL of urea detection solution was added to both standard and sample wells. Plates were incubated for 10 min at room temperature. Plate absorbance at 430 nm was measured using a Synergy HTX plate reader (BioTek, Winooski, VT). Urea concentrations were calculated from the equation derived from the linear regression of urea standards’ absorbance values.

### Cardiac tissue RNA isolation

Cardiac tissue was isolated from 1 month, 8 month, and 18-21 month old C57BL/6 mice and placed in RNAlater. If RNA was not immediately isolated, samples were frozen at -80C until RNA was to be isolated. RNA was isolated from cardiac tissue using the Qiagen RNeasy Mini Kit using the recommended protocol for tissue RNA isolation (Qiagen, Hilden, Germany). Tissue samples were disrupted and homogenized in Buffer RLT. Tissue lysate was centrifuged for 3 minutes at full speed. Lysate supernatant was carefully pipetted off and transferred to a new microcentrifuge tube. 1 volume of 70% ethanol was added to supernatant and mixed well. 600uL of sample was then transferred to an RNeasy spin column placed in a 2mL collection tube. The spin column lid was closed and samples were centrifuged at 10000 rpm for 15 seconds. The flow through in the collection tube was discarded. The remaining 600uL of sample was transferred to the spin column and samples were centrifuged at 10000 rpm for 15 seconds, with flow through subsequently being discarded. 700uL of RW1 was then transferred to the spin column and samples were centrifuged at 10000 rpm for 15 seconds. 500uL of Buffer RPE was then added to the spin column and samples were centrifuged at 10000 rpm for 15 seconds. Flow through was discarded. 500uL of Buffer RPE was added to spin columns and samples were centrifuged at 10000 rpm for 2 minutes. Spin columns were then removed from the collection tubes and collection tube and flow through was discarded. Samples were placed into a new collection tube and centrifuged at full speed for 1 minute to remove any residual buffer from samples. Spin columns were removed from collection tubes following centrifugation and placed into a new 1.5mL collection tube. 50μL of RNAse-free water was added to each spin column and samples were centrifuged for 1 minute at 10000 rpm to elute RNA isolate from samples. Flow through was then pipetted from the collection tube back onto spin column and samples were centrifuged for 1 minute at 10000 rpm to elute any residual RNA bound to the spin column. Sample RNA concentration and purity were determined using a NanoDrop Lite spectrophotometer (Thermo Scientific, Waltham, MA). Only samples with A260/80 ratios within 2.0 ± 0.10 were considered sufficiently purified for subsequent cDNA synthesis and qRT-PCR.

### Macrophage RNA isolation

Primary murine bone marrow-derived macrophages were isolated from C57BL/6 mice as previously described. Cells were cultured and treated as previously described, after which cell RNA was isolated using RNeasy Mini Kit (Qiagen, Hilden, Germany) protocol for cell monolayer RNA isolation. Briefly, cells were lysed by pipetting 600 μL of Buffer RLT in each well and using a sterile cell scraper to disrupt the cell monolayer. Six hundred microliters of 70% ethanol was added to each lysate and mixed well by pipetting. Seven hundred microliters of sample volume was then transferred to an RNeasy spin column placed in a 2 mL microcentrifuge collection tube and centrifuged for 15 seconds at 8000g. Flow through in the collection tube was discarded and remaining volume of sample lysate was transferred to the spin column and was centrifuged for 15 seconds at 8000g. Flow through in collection tube was discarded. Seven hundred microliters of Buffer RW1 was added to RNeasy spin columns and was centrifuged for 15 seconds at 8000g after which any flow through was discarded. Then, 500 μL of Buffer RPE was added to the RNeasy column and centrifuged for 15 seconds at 8000g. The flow through was discarded and 500 μL of Buffer RPE was added again to each spin column. Samples were centrifuged for 2 minutes at 8000g to wash column and ensure all residual ethanol is removed. Flow through was discarded and RNeasy spin columns were placed in a new 1.5 mL collection tube. Thirty microliters of RNAse-free water was pipetted directly onto the spin column and centrifuged for 1 minute at 8000g to elute RNA on spin column. Eluate in collection tube was then pipetted back onto column and centrifuged for 1 minute at 8000g to elute any residual RNA and concentrate sample. Sample RNA concentration and purity were determined using a NanoDrop Lite spectrophotometer (Thermo Scientific, Waltham, MA). Only samples with A260/80 ratios within 2.0 ± 0.10 were considered sufficiently purified for subsequent cDNA synthesis and qRT-PCR.

### Macrophage and cardiac RNA qRT-PCR

Sample RNA was mixed well by pipetting prior to cDNA synthesis. Total mass of RNA utilized for cDNA synthesis reaction was normalized to least concentrated RNA sample in order to ensure consistent cDNA synthesis across samples. Samples which were more concentrated were diluted with appropriate volume of RNAse-free water to normalize RNA mass used in cDNA reaction. Nine microliters of RNA or RNA diluted in RNAse-free water were added to microcentrifuge tubes. One microliter of 20X RT Enzyme Mix and 10 μL of 2X RT Buffer Mix from High Capacity RNA-to-cDNA kit (Applied Biosystems, Foster City, CA) was added to each tube. Sample tubes were prepared on ice. When ready for cDNA synthesis, sample tubes were placed in Veriti thermocycler (Applied Biosystems, Foster City, CA). cDNA reaction was catalyzed by thermocycle of 37° C for 60 minutes followed by 95° C for 5min and final rest temperature of 4° C. cDNA was stored at -20° C until ready for qRT-PCR. For the qRT-PCR reaction, 1 μL of sample cDNA was added to 8uL of RNAse-free H_2_O, 10uL of TaqMan gene expression master mix (Applied Biosystems, Foster City, CA), and 1 μL of TaqMan primer in a MicroAmp Optical 96-well reaction plate (Applied Biosystems Ref. 43067, Foster City, CA) on ice. Plate wells were sealed with MicroAmp Optical Adhesive Film (Applied Biosystems, Foster City, CA) and centrifuged at 300g for 4 minutes at 4° C. qRT-PCR reaction was catalyzed and Ct values were obtained using a QuantStudio 3 RT-PCR system (Applied Biosystems, Foster City, CA). Relative gene expression was determined by using the double delta C_t_ method of gene expression relative to housekeeping control gene, GAPDH. Primers chosen for gene expression quantification were previously reported targets of downstream Th1 and Th2 signaling pathways in macrophages: *Nos2* (Mm00440502_m1), *Il1b* (Mm00434228_m1)*, Il6* (Mm00446190_m1)*, Tnfa* (Mm00443258_m1)*, Tgfb* (Mm01178820_m1)*, Sod3* (Mm01213380_s1)*, Arg* (Mm00475988_m1) (Thermo Scientific).

### Statistics

All statistical analysis was performed using IBM SPSS statistical analysis package (IBM, Armonk, NY). A two-way ANOVA was used to assess the significance of predictor variables gel stiffness and age of cardiac extracellular matrix for dependent variables of either media nitrite concentration or urea concentration in Figures 3, 4, respectively. Tukey’s post-hoc testing was performed to assess the significance of inter-group differences. In the any case where a significant interaction term was identified from two-way ANOVA results, comparisons of interest were defined *a priori* and independent samples t-tests were leveraged to assess significance of variable interactions defined *a priori* against p-value adjusted with Bonferroni correction to account for multiple comparisons. For qRT-PCR experimental data which exhibited non-normal distributions, a Kruskal-Wallis one-way ANOVA was used to assess the significance of the predictor variable age of cardiac extracellular matrix coating. Post hoc comparisons were then performed pairwise to further elucidate significant difference between groups.
